# Supplementary material for: WordCommentsAnalyzer: A windows software tool for qualitative research
Source: F1000Res. 2018 Sep 4;7:536. Originally published 2018 May 3. [Version 2] doi: 10.12688/f1000research.14819.2 (PMC6137411; doi:10.12688/f1000research.14819.2)
Supplement: Supplementary file 3 [file f1000research-7-17614-s0002.tgz › b2a79496-4ada-47c4-9682-a99ce867836d.zip!/Eight Iranian Health Journals/Yafteh _63.docx]

© Yafteh  . This work is licensed under CC BY 4.0 (https://creativecommons.org/licenses/by/4.0/).

Volume & Issue: دوره 5 - شماره 2

Title: تأثیر مصرف نان کامل حاصل از گندم غنی شده با کودهای میکروبر شاخصهای خونی آهن

http://yafte.lums.ac.ir/article-1-970-fa.html

مقدمه : کم خونی ناشی از فقر آهن یکی از مهمترین و شایعترین مشکلات تغذیه ای دنیاست .این مطالعه به منظور تعیین اثر مصرف نان کامل حاصل از گندم غنی شده با کودهای میکرو بر شاخصهای خونی آهن انجام شد. مواد و روشها : این تحقیق بصورت کارآزمایی صحرایی و بصورت دوسوکور بر روی دو روستای تجرق و خانقاه (به عنوان تجربی و شاهد) از توابع شهرستان میانه در استان آذربایجان شرقی انجام گرفت. تعداد 350 نفر از گروههای مختلف سنی و جنسی از هر روستا براساس نمونه گیری تصادفی انتخاب شدند. در مرحله اول خونگیری cc5 خون از هر فرد اخذ شد. 257 نفر از روستای تجربی و 268 نفر از روستای شاهد در این مرحله شرکت نمودند. بررسی مصرف غذایی با روش 24 ساعت یادآمد (یک روز) و دو روز ثبت مواد غذایی برای هرفرد انجام شد.آزمایشات Hb (هموگلوبین)، آهن،TIBC (ظرفیت باند شدن آهن)، فریتین برای هر یک از نمونه ها بعمل آمد. درصد اشباع ترانسفرین نیزمحاسبه گردید. گندم حاصل از خاک غنی شده با کودهای میکرو به آرد کامل تبدیل گردید و به مدت 4 ماه به اهالی روستای تجربی داده شد. روستای شاهد نیز آرد کامل حاصل از گندم معمولی را در همین مدت دریافت نمودند. سپس همان شاخصهای ذکر شده اندازه گیری شد. تعداد افراد شرکت کننده در این مرحله 160 نفر در روستای تجربی و 152 نفر در روستای شاهد بود. برای مقایسه یافته های بین گروههای تجربی و شاهد (در هر گروه سنی) از آزمون Independent t test و برای مقایسه یافته ها در آغاز و پایان مطالعه از paired t test استفاده شد. در مورد فریتین سرم از تستهای غیر پارامتری استفاده شد. یافته ها : نتایج در گروههای مختلف سنی بصورت زیر بود: تفاوت معنی داری بین مصرف نان و دریافت مواد مغذی در هر دو روستا دیده نشد.Hb در گروه سنی 12- 5 سال روستای تجربی و شاهد نسبت به آغاز مطالعه افزایش معنی داری داشت (05/0>p)؛ ولی در سایر گروههای سنی تغییر معنی داری مشاهده نشد. درصد اشباع ترانسفرین در همه گروههای سنی روستای تجربی افزایش معنی داری داشت (05/0>p ). این مقادیر در پایان ماه چهارم در روستای تجربی نسبت به روستای شاهد تفاوت معنی داری داشت (05/0>p). در روستای شاهد گروه سنی 12 و 13 سال حتی کاهش درصد اشباع ترانسفرین را نشان داد (05/0>p ). شاخص فریتین سرم در همه گروههای سنی روستاهای تجربی و شاهد کاهش معنی داری داشت ( 01/0>p ). نتیجه گیری : بجز در گروه سنی 12-5 سال تفاوت معنی داری در نتایج حاصل از دو نوع گندم بدست نیامد. مصرف هر دو نوع گندم نیز باعث کاهش فریتین سرم شد. برای اظهار نظر در مورد مصرف این نوع گندم نیاز به مطالعات بیشتری است.

Title: مسیرآناتومیک قوس رفلکس چشمک در بیماران مبتلا به

http://yafte.lums.ac.ir/article-1-971-fa.html

مقدمه : رفلکس چشمک از تست های جدید الکترودیاگنوستیک است که توسط یک قوس رفلکسی که شاخه آوران آن عصب V و شاخه وابران آن عصب VII کرانیال است ایجاد می شود. پاسخ های این رفلکس بصورت امواج R1 و R2 و R2C است. محل آناتومیک مراکز این رفلکس در مغز هنوز بطور کامل مشخص نشده است و در مطالعات مختلف نتایج متفاوتی گزارش شده است؛ از جمله در استروک های نیمکره ای، اختلال در جزء R2 به تنهایی و یا اختلال در هر دو جزء R1 و R2 (هم بصورت یک طرفی و هم بصورت دو طرفی) گزارش شده است . هدف از انجام این مطالعه، بررسی مسیر آناتومیک قوس رفلکس چشمک بود. مواد و روشها : در این مطالعه ، بیست بیمار مبتلا به سکته مغزی نیمکره ای در طول 5/1 سال مورد بررسی قرار گرفتند و با 14 بیمار کنترل سالم( با سن و جنس مشابه) مقایسه شدند. رفلکس چشمک با روشی که بوسیله هوکیمررا توصیه شده با استفاده از یک دستگاه Toennis multilinear، اندازه گیری شد.در هر دو گروه رفلکس چشمک با شرایط مشابه انجام شد و نتایج آنها با یکدیگر مقایسه گردید. یافته ها : رفلکس چشمک در 19 مورد غیرطبیعی و در یک مورد نرمال بود. پاسخ R1 در 18 بیمار طبیعی و در 2 مورد طولانی بود. پاسخ R2 در 18 مورد بدست نیامد و یا طولانی بود که در 15 مورد بصورت دوطرفی مختل بود. در دو مورد پاسخ R2 طبیعی بود. شایعترین ریسک فاکتور در بیماران مورد مطالعه هیپرتانسیون و بعد از آن دیابت ملیتوس بود. نتیجه گیری : رفلکس چشمک در 95% بیماران غیرطبیعی بود و اختلال جزء‌ R2 در 90% بیماران مشاهده گردید که در 75% بصورت دوطرفی بود. نتیجه گیری نهایی این بود که اگر چه در بیماران مبتلا به سکته های نیمکره ای ممکن است اختلال رفلکس چشمک هم در موج R1 و هم در موج R2 مشاهده شود؛ ولی بیشترین الگوی اختلال، بدست نیامدن یا طولانی شدن موج R2 بصورت دو طرفی می باشد.

Title: بررسی اثر داروی دانوروبیسین به تنهایی و به همراه سایمتیدین بر سلولهای بنیادی مغز استخوان موش

http://yafte.lums.ac.ir/article-1-972-fa.html

مقدمه: خونسازی یک سیستم پویا بوده و در مغز استخوان انجام می شود .تعداد محدودی از سلولهای مغز استخوان را سلولهای بنیادی خونساز تشکیل می دهند که شامل سلولهای بنیادی اولیه ، سلولهای پیش ساز و سلولهای پیشتاز می باشند. سلولهای بنیادی اولیه دارای توانایی ایجاد کلونی در محیط کشت ( CFU-C) و طحال موش اشعه دیده ( CFU-S) هستند. آسیب دیدن این سلولها می تواند سبب نوتروپنی ، آگرانولوسیتوز ، ترومبوسیتوپنی ، پان سیتوپنی یا آنمی آپلاستیک شود . یک سلول خونساز وقتی زنده و فعال به حساب می آید که توانایی تکثیر و ایجاد کلونی را داشته باشد و در غیر اینصورت عملا سلولی مرده به حساب خواهد آمد. سنجش عملکرد سلولهای بنیادی توسط دو سیستم in vivo و in vitro انجام شده که سیستمهای in vivo ارجح تر می باشند. اغلب داروهای ضد سرطان دارای آثار مخرب بر سلولهای خونساز مغز استخوان هستند و نوتروپنی عمومی ترین عامل محدود کننده استفاده از شیمی درمانی است. مواد و روشها : در تحقیق حاضر حدود 150 سر موشBlab/c نر سفید خریداری شده از موسسه رازی مورد استفاده قرار گرفت.همچنین از دانوروبیسین که داروی مهمی در درمان لوسمی میلوئید حاد (AML) است ، به عنوان عامل آسیب رسان به مغز استخوان و از سایمتیدین به عنوان عامل محافظ مغز استخوان در مقابل این دارو استفاده شد. در این تحقیق روش سنجش کلونی طحال مورد استفاده قرار گرفت.اساس این روش تزریق سلولهای مغز استخوان به موشهایی است که دوز کشنده اشعه گاما را دریافت نموده اند. 10-8 روز پس از تزریق سلولهای خونساز جدا شده از مغز استخوان ، هر سلول بنیادی در طحال موش اشعه دیده یک کلونی ایجاد می کند .تعداد کلونیها شمارش شده و سپس نسبت بقاء (SF) مورد آنالیز آماری قرار گرفت. یافته ها : توسط روش سنجش کلونی طحال نشان داده شدکه سایمتیدین با غلظت 15 میلی گرم بر کیلوگرم می تواند نسبت بقای سلولهای مغز استخوان موش را در مقابل دانوروبیسین در غلظتهای 5 و 10 و 20 میلی گرم بر کیلو گرم افزایش دهد . احتمالا سایمتیدین توسط جمع آوری رادیکال های آزاد و از طریق مهار سیتوکروم P450 و جلوگیری از متابولیسم کبدی گلوتاتیون و نیز با مهار اثر هیستامین و تعدیل سیستم ایمنی اثر محافظتی خود را اعمال می نماید. نتیجه گیری : سایمتیدین قادر است اثر سایتولتیک و سایتوتوکسیک دانوروبیسین را بر روی سلولهای بنیادی مغز استخوان موش کاهش دهد.

Title: بررسی اثر توأم ATP-MgCl2 با محلولهای هیپرتونیک در احیاء شوک هموراژیک دررات

http://yafte.lums.ac.ir/article-1-973-fa.html

مقدمه : در سالهای اخیر کارا یی محلولهای هیپرتونیک در احیا, شوک هموراژیک در مطالعات آزمایشگاهی وبررسی های کلینیکی نشان داده شده است. ازطرفی تجویز ATP-MgCl2 نیزدر درمان شوک هموراژیک جهت بهبود برون ده قلبی, کارایی قلب ,پرفوزیون بافتی بکار می رود. در این تحقیق اثرات محلولهای هیپرتونیک همراه با محلول ATP-MgCl2در درمان شوک هموراژیک رات بررسی شد. مواد و روشها : در این مطالعه تجربی 21 رات نر از نژاد ویستار به طور تصادفی درسه گروه قرار گرفتند.پس از بیهوش نمودن, شوک هموراژیک بوسیله خونریزی ایجاد وحیوانات بمدت 30دقیقه در حالت شوک نگهداری شدند. سپس به گروه اول محلول هیپرتونیک کلرور سدیم 5%،به گروه دوم ATP-MgCl2 و به گروه3 هر دو محلول تزریق شد. فاکتورهای فشارخون ،الکترولیتهای سرم(سدیم و پتاسیم)،هماتوکریت،پروتئین توتال، آلبومین،ضربان قلب، اسمولالیته سرم و ادرار قبل، ,بلافاصله بعد از شوک ویک ساعت پس از تزریق محلولها اندازه گیری شد. فشار خون با استفاده از دستگاه فیزیوگراف و سدیم و پتاسیم با فلم فتومتر و سایر فاکتورهای خونی توسط کیت های مخصوص اندازه گیری شد. همچنین اسمولالیته سرم و ادرار توسط دستگاه اسمومتر اندازه گیری شد. یافته ها : میانگین غلظت سدیم یکساعت بعد از احیا، در گروه 1و3 نسبت به گروه 2 افزایش معنی داری داشت (05/0p<) ومیانگین غلظت آلبومین سرم در گروه 2 نسبت به گروههای دیگر افزایش معنی داری از خود نشان داد (05/0p<).از نظر اسمولالیته سرم وادرار در سه گروه تفاوت معنی داری وجود نداشت.میانگین فشار خون سیستولی در 15 دقیقه اول بعد از احیا, در گروه 2 کاهش معنی داری را نسبت به گروه 1و3 از خود نشان داد (05/0p<) و در بین گروه 1و3 ,گروه 3 افزایش بیشتری داشت هر چند که اختلاف معنی دار نبود. نتیجه گیری : اگر چه تغییراتی در غلظت سدیم یا آلبومین پلاسما بعد از درمان در گروه 2 نسبت به سایر گروهها مشاهده شدو نیزدر گروه سه در 15 دقیقه اول بعد از احیاء موفق شد که فشار خون را از دیگر گروهها بالاتر ببرد؛ ولی نتوانست در پایان احیاء، فشار خون را در آن محدوده حفظ کند، به نظر میرسد محلول ATP-MgCl2 به تنهایی و توام آن با محلول هیپرتونیک در مقایسه با محلول هیپرتونیک به تنهایی اثرات مفیدی را در بهبود پارامترهای همودینامیک و آزمایشگاهی در احیاء شوک نداشته باشد.

Title: بررسی عوامل باکتریال مننژیت کودکان و تعیین نتایج آنتی بیوگرام

http://yafte.lums.ac.ir/article-1-974-fa.html

مقدمه : مننژیت باکتریال هنوز به عنوان یکی از خطرناکترین بیماری های عفونی شناخته شده است که در صورت عدم تشخیص و درمان به موقع، با عوارض و تلفات زیادی همراه خواهد بود. هدف از این مطالعه، بررسی شایع ترین عوامل باکتریایی مننژیت کودکان زیر ده سال و تعیین مقاومت دارویی باکتری های جدا شده در مراجعین به بیمارستان های آموزشی شهر همدان بود. مواد و روشها: در طی دوسال (از خرداد ماه سال 77 لغایت اردیبهشت سال 79) تعداد 582 کودک مشکوک به مننژیت از نظر کشت مایع نخاع، توزیع سنی، جنسی، فصلی و نتایج آنتی بیوگرام مورد بررسی قرار گرفتند.اطلاعات ضروری بیماران در پرسشنامه تنطیمی درج و سپس توسط نرم افزار آماری Epi-6 مورد تجزیه و تحلیل قرار گرفتند. گونه های میکروبی با روشهای بیوشیمیایی و سرولوژیک شناسایی شدند. برای آزمایش آنتی بیوگرام نیز از روش دیسک دیفیوژن کربی-بائر استفاده شد. یافته ها : از مجموع 582 کودک زیر ده سال مشکوک به مننژیت تنها 46 کودک (9/7%) دارای کشت مثبت باکتریایی بودند که از این تعدد 9/58% باکتریهای گرم مثبت و 1/41% باکتریهای گرم منفی جدا شد. شایع ترین باکتریهای جدا شده عبارت بودند از: استرپتوکوک پنومونیه 9/23% ، استافیلوکوک اورئوس 1/13%، اشریشیا کلی 9/10%، نایسریا مننژیتیدیس و پسودوموناس آئروژینوزا هرکدام 5/6%. بیشترین میزان بروز عفونت (3/28%) در کودکان با گروه سنی 2 -0ساله (3/28%) ، جنس مذکر (4/54%) و در فصل پائیز (7/43%) بود. در مورد نتایج آنتی بیوگرام از هشت آنتی بیوتیک تست شد، موثرترین آنتی بیوتیک برای هر دو گروه باکتری گرم مثبت و منفی جدا شده از بیماران، سفتی زوکسیم، کانامایسین و جنتامایسین بود؛ اما نسبت به آموکسی سیلین، آمپی سیلین و کلرامفنیکل مقاومت نسبتا" بالایی وجود داشت. نتیجه گیری: با توجه به نتایج بدست آمده در این مطالعه، می توان چنین نتیجه گیری نمود که باکتری های گرم مثبت بویژه استرپتوکوک پنومونیه و استافیلوکوک اورئوس از عوامل اصلی ایجاد کننده مننژیت باکتریال کودکان منطقه مورد بررسی بشمار میآیند. اکثر گونه ها نسبت به آنتی بیوتیک های رایج مانند آموکسی سیلین، آمپی سیلین و کلرامفنیکل مقاومت نسبتا" بالایی داشتند.

Title: مقاومت به آنتی بیوتیک در عفونت ادراری کودکان بستری در بیمارستان

http://yafte.lums.ac.ir/article-1-975-fa.html

مقدمه : عفونت ادراری از بیماری های شایع باکتریال دوران کودکی است. حدود 5-3% دختران و 1% پسران مبتلا به عفونت ادراری می شوند. درمان عفونت ادراری به واسطه افزایش شیوع سوشهای مقاوم، مشکل و درمان تجربی با آنتی بیوتیک های رایج مثل آمپی سیلین، کوتریموکسازول و آموکسی سیلین منجر به ایجاد مقاومت بیشتر و صرف هزینه های بیشتر شده است. هدف ما از این مطالعه بررسی مقاومت آنتی بیوتیکی در بیماران زیر 14 سال با کشت مثبت ادرار بر اساس نتیجه آنتی بیوگرام بود. مواد و روشها : در این مطالعه توصیفی ـ مقطعی کلیه بیمارانی که در طی یکسال (81-80) با عفونت ادراری به بیمارستان کودکان خرم آباد مراجعه کرده بودند (127 نفر)، مورد بررسی قرار گرفتند. روش نمونه گیری برای انجام کشت و آنتی بیوگرام در بیماران واجد کنترل ادراری، گرفتن نمونه وسط ادرار و در اطفال بدون کنترل، اسفنکتری، آسپیراسیون سوپراپوبیک بود. یافته ها : بر اساس یافته های آزمایشگاهی، ارگانیسم رشد کرده در کشت ادرار مبتلایان به این شرح بود: اشرشیاکولی (3/73%) ، پروتئوس (6/12%) ، کلبسیلا (4/9%) انتروباکتر (1/3%)، سیتروباکتر (8/0%) و یرسینیا (8/0%). در مجموع مقاومت آنتی بیوتیکی در بیماران 9/96% بود و میزان مقاومت میکروارگانیسم ها به آنتی بیوتیک های کشت شده به این شرح بود: آمپی سیلین (2/88%) ، آموکسی سیلین (6/86%)، کوتریموکسازول (6/75) نیتروفورانتوئین (9/18%)، نالیدیکسیک اسید (2/10%)،سفی کسیم (7/4%) سفتریاکسون (1/3%)، جنتامایسین (8/11%)، آمیکاسین (1/3%)، سیپروفلوکساسین (7/4%). نتیجه گیری : نتایج نشان داد میزان مقاومت به آنتی بیوتیک های خوراکی که به طور رایجی در سایر عفونت ها تجویز می شوند بارز تراست.

Title: میزان شیوع انگل های روده ای در کارگران نانوایی های شهرخرم آباد در سال 1380

http://yafte.lums.ac.ir/article-1-976-fa.html

مقدمه : در کشور ما از دیرباز مسئله انگل مورد توجه مردم و پزشکان بوده است و در حال حاضر بیماری های انگلی در جامعه ما هنوز از مسائل مهم بهداشتی است.هدف از انجام این مطالعه بررسی میزان شیوع انگل های روده ای در کارگران نانوایی شهر خرم آباد بود. مواد و روشها : در این مطالعه کلیه نانوائی های شهر خرم آباد به تعداد 270 مورد و تمامی کارگران شاغل در نانوائی ها به تعداد 933 نفر به صورت سرشماری انتخاب شدند و مدفوع آنها به روش فرمل – دترجنت مورد آزمایش قرار گرفت. یافته ها : نتایج نشان داد که فراوانی کلی انگل های روده ای 2/13 درصد است. از این تعداد 4/12% به یک انگل و 8/0% به دو نوع انگل آلوده بودند. بیشترین فراوانی در عفونت با تک یاخته ها مربوط به ژیاردیا(9/7% ) بود. یافته ها بیانگر این بود که افراد با سطح سواد بالا دارای میزان آلودگی انگلی روده ای کمتری هستند. همچنین اگر چه آگاهی از راه انتقال بیماری باعث کمتر شدن شیوع آلودگی می شود اما این تفاوت از نظر آماری معنی دار نبود. از نظر میزان آلودگی , بین دو گروه دارای کارت تندرستی و گروه فاقد کارت تندرستی نیز تفاوت معنی داری مشاهده نشد. نتیجه گیری : جهت کاهش آلودگی در این افراد, مواردی مثل آزمایش مدفوع هر سه ماه یکبار با روش های برتر تغلیظی، نظارت و اعمال ضوابط و مقررات دقیق بهداشتی از سوی کارشناسان بهداشت، آموزش های مستقیم در مورد بهداشت فردی و اجتماعی و آموزش نحوه انتقال انگل ها پیشنهاد می شود.

Title: ارزیابی روشهای تشخیص و مکان یابی غده های مهاجم در بیماران پاراتیروئیدکتومی شده در دو بیمارستان دانشگاه علوم پزشکی تهران طی ده سال (1377-1368)

http://yafte.lums.ac.ir/article-1-977-fa.html

مقدمه : تشخیص غده های مهاجم و یافتن مکان آنها در بیماران دچار هیپرپاراتیروئیدیسم موضوعی است که همیشه مورد توجه جراحان بوده است. تا دو دهه پیش این کار صرفاً با جستجوی کامل گردن توسط جراح امکان پذیر بود؛ اما در دهه های اخیر با عرضه شدن روشهای تصویربرداری بهتر نظیر سونوگرافی پیشرفته و اسکن های ایزوتوپیک پیشرفت قابل ملاحظه ای در این زمینه حاصل شده است. برای ارزیابی این روشها مطالعه ای طراحی و اجراء گردید. مواد و روشها : این یک مطالعه توصیفی گذشته نگر در بیماران مبتلا به هیپرپاراتیروئیدیسم اولیه است که طی سالهای 1368 تا 1377 در بیمارستانهای امام خمینی و دکتر شریعتی دانشگاه علوم پزشکی تهران تحت عمل جراحی پاراتیروئیدکتومی قرار گرفته اند. هدف از مطالعه ارزیابی حساسیت (sensitivity) و صحت (Accuracy ) تشخیص و تعیین محل غده (های) مهاجم توسط تجهیزات تصویربرداری موجود در مقایسه با یافته های حین عمل است. یافته ها : 61 بیمار واجد شرایط مطالعه بودند (11 نفر مرد 18% و 50 نفر زن 82%). 47 مورد آدنوم منفرد (77%)، دو مورد آدنوم متعدد (3%) و 12 مورد هیپرپلازی (20%) گزارش شده بود. اسکنMIBI برای 24 بیمار انجام شده بود. حساسیت و صحت آن برای آدنوم منفرد به ترتیب 70% و 100% بود.نتیجه اسکن MIBI در تمام بیماران دچار هیپرپلازی و آدنوم متعدد که مورد بررسی قرار گرفتند،منفی کاذب بود. حساسیت اسکن Th-Tc برای آدنوم منفرد 3/33% بود. حساسیت سونوگرافی برای آدنوم 5/71% و برای هیپرپلازی 40% محاسبه شد. صحت تشخیص در هر دو مورد 100% بود.حساسیت و صحت تشخیص جراح، در حین عمل، برای آدنوم بترتیب 100% و 96% و برای هیپرپلازی 3/83% و 100% بود نتیجه گیری : بر اساس نتایج بدست آمده قضاوت جراح با تجربه در تشخیص و تعیین محل غده (های) مهاجم در هیپرپاراتیروئیدیسم اولیه حساسیت و صحت بالاتری از نتایج تصویر برداری های موجود دارد که بخصوص در آدنوم متعدد و هیپرپلازی صدق می کند. بنابراین توصیه می گردد، هنوز هم به هنگام جراحی هیپرپاراتیروئیدیسم، تمام غدد بررسی شده و از جستجوی محدود با اتکا به یافته های تصویر برداری خود داری شود. گرچه با کسب تجربه روز افزون و عرضه تکنولوژیهای جدید نظیر( Radiotracing ) می توان هر روز انتظار نتایج بهتری از تصویر برداری داشت.

Title: سنجش سطح سرمی (CRP) C-Reactive Protein در تعیین

http://yafte.lums.ac.ir/article-1-978-fa.html

مقدمه : بیماری عروق کرونر از شایع ترین، جدی ترین، مزمن ترین و کشنده ترین بیماریها در جهان است. این گروه بیماریها شامل طیفی از بیماریهاست که یک سر آن انفارکتوس حاد و سر دیگر آن ایسکمی بدون علامت است. CRP در تعیین پیش آگهی آنژین صدری ناپایدار و انفارکتوس حاد قلبی اهمیت خاص دارد. هدف از انجام این مطالعه سنجش سطح سرمی CRP در تعیین پیش آگهی بیماران مبتلا به سندرمهای حاد کرونری بود. مواد و روشها : در این مطالعه مقطعی، بیماران بستری در بیمارستانهای شهر خرم آباد با تشخیص آنژین صدری ناپایدار (32 نفر) و انفارکتوس حاد قلبی (32 نفر) با افراد سالم (32 نفر) مقایسه شدند. کلیه یافته های کلینیکی و آزمایشگاهی در هنگام بستری، 12 ساعت بعد، 48 ساعت بعد و هنگام ترخیص جمع آوری شد. تمام بیماران برای 14 روز پس از ترخیص پیگیری شدند. یافته ها : در بیماران مبتلا به انفارکتوس حاد قلبی میزان CRP بسیار بالاتر از بیماران مبتلا به آنژین صدری ناپایدار بود. وقوع حوادث قلبی در طی 14 روز پس از ترخیص با میزان CRP در 48 ساعت اول ارتباط معنی دار داشت (02/0p=). میزان CRP با کسر تخلیه بطنی ارتباط معکوس داشت (44/0-r=). نتیجه گیری : بر اساس این مطالعه میزان CRP در بیماران انفارکتوس حاد قلبی بالاتر از بیماران با آنژین صدری ناپایدار بود و بالا بودن CRP با وقایع خارج بیمارستان در طی 14 روز پیگیری ارتباط داشت..

Title: ترومای نافذ قلب: پنج مورد پارگی حفرات قلب در بیمارستان شهدای عشایر خرم آباد طی یکسال (1382-1381)

http://yafte.lums.ac.ir/article-1-979-fa.html

مقدمه : تروما یکی از عوامل اصلی مرگ و میر، مخصوصاً در جوامع شهری است و در این میان صدمات قلبی از جمله مهمترین موارد آن به شمار می رود. وضعیت علائم حیاتی بیمار در بدو ورود ، مکانیسم صدمه، وجود تامپوناد قلبی از عوامل موثر بر پیش آگهی می باشند. چون بسیاری از این مجروحین (70%) که زنده به مراکز درمانی می رسند، علائم دال بر وجود صدمه قلبی را از خود نشان نمی دهند؛ لذا تشخیص سریع مبتنی بر علائم بالینی و نیز استفاده مناسب از روشهای تشخیصی (اکوکاردیوگرافی دو بعدی ـ پونکسیون مایع پریکارد ـ کاتتراسیزیون قلبی) در صورت ثابت ماندن علائم حیاتی بیمار از جمله مهمترین راههای تشخیصی است که در نجات جان بیماران موثر خواهد بود. انجام عمل جراحی و ترمیم زخم قلب در تمام مجروحین به عنوان تنها روش قابل قبول درمانی پیشنهاد می گردد. معرفی موارد : در این مطالعه تعداد پنج بیمار که در فاصله کمتر از یکسال (دی ماه 81 لغایت آبان ماه 82) به اورژانس بیمارستان شهدای عشایر خرم آباد ارجاع داده شده بودند، مورد بررسی قرار گرفتند. این بیماران در سه مرحله، داخل اورژانس، حین عمل جراحی، و از نظر عوارض بعد از عمل مورد بررسی قرار گرفتند. یک مورد از این بیماران (دختر بچه 8 ساله) به طور تصادفی دچار صدمه با ساچمه شده بود و 4 مورد دیگر، همگی توسط ضربه چاقو دچار صدمه شده بودند. محل صدمه در 3 بیمار کناره چپ استرنوم بود. یک مورد محل صدمه کناره راست استرنوم و در یک مورد زیر زانو گزیفونید بود. محل پارگی قلب در دو مورد بطن چپ، دو مورد بطن راست و در یک مورد دهلیز راست بود. در 3 مورد عوارض بعد از عمل وجود نداشت. در یک مورد بلوک شاخه ای راست قلب و در یک بیمار ضعف در عضلات دست راست ناشی از صدمه به شبکه بازویی وجود داشت.
